# *Baeckea frutescens* Suppresses Melanogenesis via Modulation of PKA/CREB and ERK/MAPK Pathways: Insights from Cellular, Zebrafish, and In Silico Analyses

**DOI:** 10.3390/molecules31101685

**Published:** 2026-05-16

**Authors:** Chih-Li Yu, Yen-Li Huang, Yingying Huang, Yu Zhong, Haiyue Pang, Guey-Horng Wang

**Affiliations:** 1 Engineering Research Center of Natural Cosmeceuticals College of Fujian Province, Department of Public Health and Medical Technology, Xiamen Medical College, Xiamen 361023, China; 202000051180@xmmc.edu.cn (C.-L.Y.); 15289867182@163.com (Y.Z.); 2 Department of Post-Baccalaureate Veterinary Medicine, Asia University, Taichung 413305, Taiwan; ellis7374365@asia.edu.tw

**Keywords:** *Baeckea frutescens*, melanogenesis, molecular docking, zebrafish

## Abstract

Background: *Baeckea frutescens* L. (BF) has been reported as a potential natural source for skin-whitening agents. However, its antimelanogenic activity and mechanisms remain unclear. Methods: The antimelanogenic effects of BF were evaluated in α-melanocyte-stimulating hormone (α-MSH)-stimulated B16F10 cells and in zebrafish embryos. Cell viability, intracellular tyrosinase activity and melanin content were measured. Western blot (WB) analysis was used to examine melanogenesis-related proteins. Network pharmacology and molecular docking were performed to predict potential targets and interactions of BF-derived metabolites. Results: The ethanolic extract of BF reduced intracellular tyrosinase activity and melanin content in cells without cytotoxicity. Western blot analysis showed decreased expression of microphthalmia-associated transcription factor (MITF) and its downstream melanogenic enzymes, including tyrosinase (TYR), tyrosinase-related protein-1 (TRP-1), and dopachrome tautomerase (DCT). In addition, BF reduced phosphorylation of protein kinase A (PKA), cAMP responsive element-binding protein (CREB) and extracellular signal-regulated kinase (ERK), suggesting potential suppression of PKA/CREB and ERK signaling pathways. These regulatory effects may contribute to MITF downregulation and subsequent inhibition of melanogenesis. BF reduced melanin accumulation in zebrafish embryos. Network pharmacology and molecular docking analyses further suggested that BF-derived metabolites, particularly bayogenin, may interact with multiple melanogenesis-related targets. Conclusions: BF may inhibit melanogenesis through coordinated modulation of multiple signaling pathways and may represent a promising skin-whitening candidate.

## 1. Introduction

Skin whitening has garnered increasing attention, particularly in African and Asian countries [1]. Melanin serves as a natural photoprotective pigment from ultraviolet (UV) damage. However, excessive production of melanin leads to hyperpigmentation and uneven skin tone. The pharmaceutical and cosmetic products for skin-whitening have grown rapidly. Nevertheless, some whitening agents can cause side effects, including cutaneous irritation and contact dermatitis [2,3,4]. Consumers’ increasing concern for ingredient safety and efficacy has driven growing interest in safer alternatives for skin whitening. Developing natural, non-toxic skin-whitening ingredients using plant extracts has become a promising research direction [5].

The catalytic oxidation of L-tyrosine to dopaquinone is performed by TYR. As the rate-limiting enzyme of melanogenesis, TYR is recognized as a key regulator of melanin production [6]. Recent research has focused on developing new compounds or plant extracts that inhibit tyrosinase activity. The TYR inhibitors may have skin-whitening effects and have potential applications in cosmetic products [7,8]. TYR is tightly regulated by multiple cellular signaling pathways, which influence its expression and enzymatic function [9].

α-MSH is a peptide hormone commonly used to induce the process of melanin production. Its action significantly upregulates the enzymatic activity of TYR and associated melanogenic proteins [10,11]. Previous studies have demonstrated that MITF is upregulated by α-MSH through several signaling pathways [12].

In addition, α-MSH stimulates intracellular cyclic adenosine monophosphate (cAMP) production, leading to activation of protein kinase A (PKA) and subsequent phosphorylation of cAMP response element-binding protein (CREB), which ultimately enhances MITF expression [13].

MAPK signaling adds further complexity to melanogenesis regulation. The MAPK signaling pathways, including c-Jun N-terminal kinase (JNK) and ERK, are involved in the regulation of MITF. In particular, JNK is a stress-responsive pathway that can enhance MITF transcriptional activity and promote melanogenic enzyme expression [14]. α-MSH also activates ERK phosphorylation, which, through the MAPK signaling pathway, also contributes to the increased expression of MITF [15].

Moreover, the interaction of α-MSH with the melanocyte-specific melanocortin-1 receptor (MC1R) promotes protein kinase B (AKT) phosphorylation, and subsequent inhibition of glycogen synthesis kinase-3β (GSK-3β) further upregulates the expression of MITF [13,16]. MITF directly regulates the expression of TYR, TRP-1 and DCT, leading to increased protein levels and melanin production [9].

Zebrafish are a widely used vertebrate model for in vivo studies due to their human-like organ systems and conserved genome sequence [17]. Importantly, the optical transparency of their embryos and larvae enables direct observation of melanogenesis, making them suitable for evaluating depigmentation effects [18].

Network pharmacology has emerged as an in silico, systems-level approach for elucidating the multi-component and multi-target mechanisms of natural products [19,20]. By integrating chemical profiling, target prediction, and pathway enrichment analysis, this approach may provide a systematic framework for exploring potential interactions between candidate metabolites and melanogenesis-related signaling pathways [21,22]. Previous studies suggest that melanogenesis is regulated by multiple signaling pathways converging on MITF [9]. Accordingly, network pharmacology may offer a framework to explore how plant-derived metabolites modulate these pathways. However, these predictions are primarily hypothesis-generating and require further experimental validation. In this context, integrating network pharmacology with in vitro and in vivo assays may help to provide a more systematic understanding of the antimelanogenic potential of BF extracts.

*Baeckea frutescens* L. is primarily distributed across tropical Asia and Australasia [23]. Recent studies showed that BF extracts exhibit anti-inflammatory, antioxidant, antibacterial, and wound healing promotion [24,25]. This study aimed to evaluate the antimelanogenic effects of BF extracts using α-MSH-stimulated B16F10 cells and zebrafish models, and to explore the underlying molecular mechanisms. Furthermore, an integrative strategy combining network pharmacology and molecular docking was employed to predict potential targets and interactions of BF-derived metabolites. This combined approach may provide a more comprehensive understanding of the antimelanogenic potential of BF and support its development as a natural skin-whitening agent.

## 2. Results

### 2.1. Mushroom Tyrosinase Inhibition and Effects of BF on Melanogenesis in B16F10 Cells

**Figure 1:**
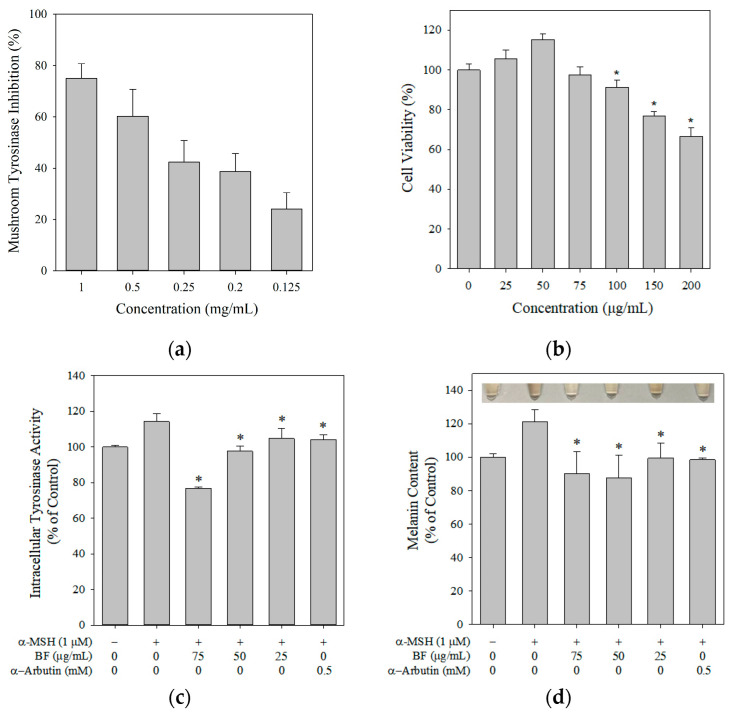
Effects of BF on (**a**) mushroom tyrosinase inhibition, (**b**) cell viability, (**c**) intracellular tyrosinase activity, and (**d**) melanin content and representative images showing pigmentation changes in each group. Data are presented as mean ± SD (*n* = 3). For panel (**b**), statistical comparisons were performed against the untreated control group. For panels (**c**,**d**), * *p* < 0.05 vs. the α-MSH group. α-Arbutin was used as a reference control in the cellular melanogenesis assays.

Mushroom tyrosinase inhibitory activity increased in a concentration-dependent manner, reaching 74.92% inhibition at 1 mg/mL (Figure 1a). The IC_50_ values of BF and ascorbic acid for mushroom tyrosinase inhibition were 0.391 ± 0.055 mg/mL, and 0.667 ± 0.051 µg/mL, respectively. Ascorbic acid was used as a reference compound to provide a comparative benchmark for the assay [26]. Mushroom tyrosinase is widely used as a preliminary screening model for evaluating potential melanogenesis inhibitors [27]. These findings indicate that BF exhibited tyrosinase inhibitory activity in this preliminary enzyme-based assay, supporting its further evaluation in cellular melanogenesis models.

Although the in vitro mushroom tyrosinase assay demonstrated that BF could inhibit tyrosinase activity, this result does not confirm its ability to suppress melanin production in cells. Cell viability was evaluated, showing that the cell viability remained above 90% at a concentration of 75 μg/mL (Figure 1b). Thus, the following experiments were performed at concentrations up to 75 μg/mL. α-MSH stimulation increased intracellular tyrosinase activity and melanin content in B16F10 cells, whereas BF treatment reduced both parameters (Figure 1c,d), consistent with Wen’s report [28].

### 2.2. Melanogenesis-Related Protein Expression

**Figure 2:**
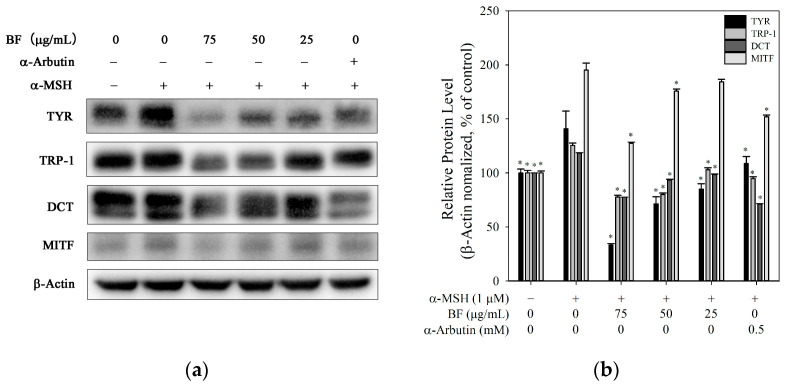
Modulation of melanogenesis-related proteins by BF in B16F10 cells. (**a**) Representative immunoblots showing the expression of TYR, TRP-1, DCT, and MITF. (**b**) Protein levels were normalized to β-actin. Data are presented as mean ± SD (*n* = 3). * *p* < 0.05 vs. α-MSH group. α-Arbutin was used as a positive control.

Based on the findings in Section 2.1, the reduction in melanin content may be influenced by changes in intracellular tyrosinase activity. To further investigate the underlying mechanism, WB analysis was performed to examine melanogenesis-related proteins. α-MSH significantly increased the protein levels of TYR, TRP-1, DCT, and MITF in B16F10 cells, whereas BF treatment inhibited their expression (Figure 2a,b). As a central regulator of melanogenesis, MITF modulates the expression of downstream enzymes, including TYR, TRP-1, and DCT, thereby influencing melanin synthesis. This suggested that BF may interfere with MITF expression, thereby decreasing downstream TYR expression and reducing melanin production.

### 2.3. BF Suppresses Melanogenesis via Modulation of PKA, CREB and ERK Signaling

**Figure 3:**
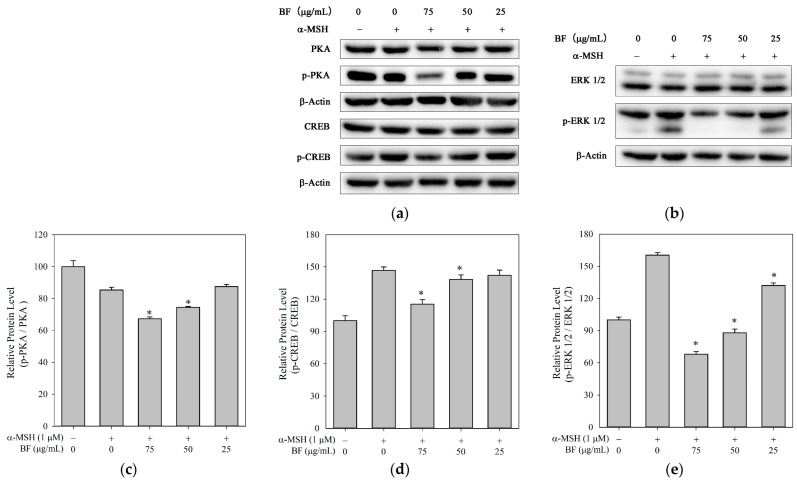
Modulation of PKA/CREB and ERK signaling by BF in B16F10 cells. (**a**) Representative immunoblots showing the expression of PKA, p-PKA, CREB and p-CREB. (**b**) Representative immunoblots showing the expression of ERK1/2, and p-ERK1/2. (**c**–**e**) Quantification of p-PKA/PKA (**c**), p-CREB/CREB (**d**), and p-ERK1/2/ERK1/2 (**e**) ratios normalized to β-actin. Data are presented as mean ± SD (*n* = 3). * *p* < 0.05 vs. α-MSH group.

The cAMP/CREB signaling pathway plays a central role in the regulation of melanogenesis. In B16F10 cells, binding of α-MSH to MC1R promoted intracellular cAMP accumulation, which released active PKA to phosphorylate CREB. Increased CREB phosphorylation regulated MITF expression, thereby promoting melanin synthesis [29]. As shown in Figure 3a,c,d, BF treatment reduced the phosphorylation levels of PKA and CREB, compared with the α-MSH group. This finding could suggest that BF inhibits melanogenesis, at least in part, through modulation of the PKA/CREB signaling pathway.

The MAPKs signaling pathway was involved in the regulation of melanin synthesis, including ERK and JNK MAPKs [6]. In Figure 3b,e, BF treatment decreased the level of ERK1/2 phosphorylation in B16F10 cells, consistent with previous reports [30,31]. In contrast, BF had no significant effect on the protein expression of JNK 1/2/3 MAPK (Figure S1). Therefore, the observed inhibition of ERK phosphorylation by BF may contribute to the downregulation of MITF and subsequent suppression of melanogenesis.

### 2.4. BF Modulated AKT/GSK-3β/β-Catenin Signaling in B16F10 Cells

The Akt/GSK-3β/β-catenin signaling pathway has been reported to participate in the regulation of melanogenesis. Akt can inhibit GSK-3β through Ser9 phosphorylation, which may stabilize β-catenin and enhance MITF-dependent melanogenesis [32].

**Figure 4:**
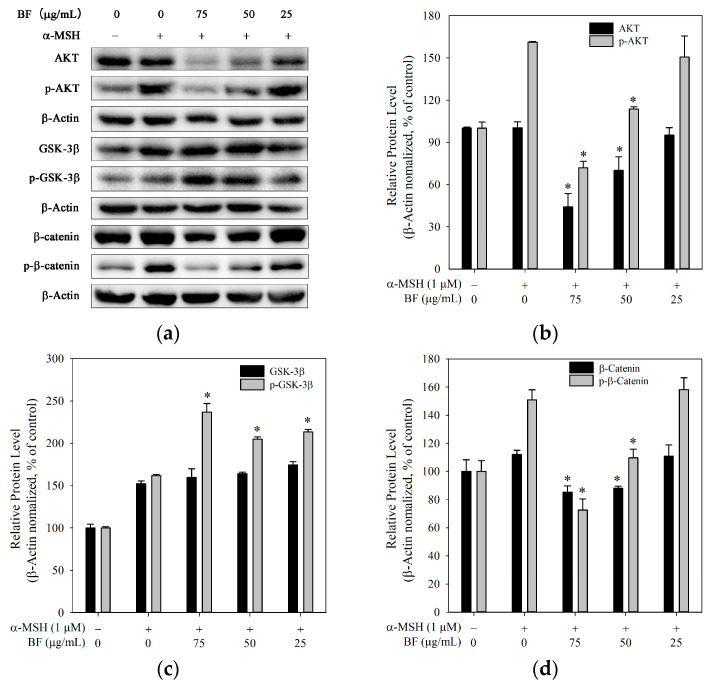
Modulation of AKT, GSK-3β, and β-catenin signaling by BF in B16F10 cells. (**a**) Representative immunoblots showing the expression of AKT, p-AKT, GSK-3β, p-GSK-3β, β-catenin, and p-β-catenin. (**b**–**d**) Quantification of AKT and p-AKT (**b**), GSK-3β and p-GSK-3β (**c**), and β-catenin and p-β-catenin (**d**) normalized to β-actin. Data are presented as mean ± SD (*n* = 3). * *p* < 0.05 vs. α-MSH group.

As shown in Figure 4a,b,d, BF treatment was associated with reduced levels of AKT and p-AKT, suggesting attenuation of upstream signaling. Consistently, total β-catenin and phosphorylated β-catenin (Ser675) levels were decreased. Since phosphorylation at Ser675 has been reported to enhance protein stability and transcriptional activity [33], the observed reduction in β-catenin may be partially attributed to decreased Ser675 phosphorylation. This may be associated with reduced MITF-dependent melanogenic gene expression, thereby decreasing melanin production.

Although p-GSK-3β (Ser9) was increased, total GSK-3β expression remained unchanged (Figure 4c), suggesting that GSK-3β may not be the sole regulator under these conditions. In addition, GSK-3β phosphorylation may be influenced by additional upstream regulatory mechanisms beyond Akt [34].

### 2.5. BF Reduced Melanin Accumulation in Zebrafish Embryos

**Figure 5:**
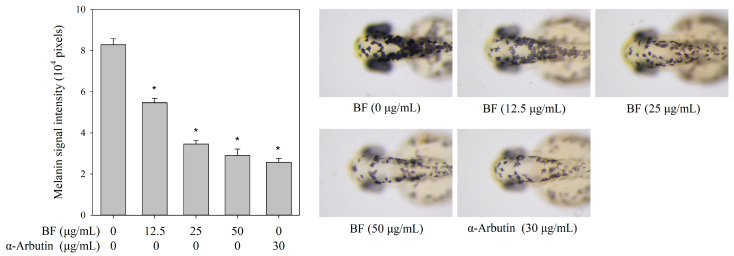
Effects of BF on melanin signal intensity in zebrafish embryos. Data are reported as mean ± SD (*n* = 10). * *p* < 0.05 vs. untreated control group. α-Arbutin was used as a positive control.

When zebrafish embryos were incubated with BF at concentrations up to 50 μg/mL, no significant effects on survival, development, or phenotypic characteristics were observed (Table S1). BF concentrations for zebrafish pigmentation assessment were selected based on these embryo toxicity results. BF treatment led to a concentration-dependent decrease in melanin signal intensity in zebrafish embryos, suggesting reduced melanin accumulation in vivo (Figure 5). Although no apparent developmental toxicity was observed, the possibility of non-specific pigmentation-related effects cannot be completely excluded. Therefore, these results should be interpreted as supportive in vivo evidence rather than definitive proof of direct melanogenesis inhibition.

### 2.6. UHPLC-Q Exactive Orbitrap-HRMS-Based Compound Profiling and Network Pharmacology Analysis

UHPLC-Q Exactive Orbitrap-HRMS analysis was performed to obtain a preliminary chemical profile of the BF ethanolic extract. To improve metabolite coverage, both positive- and negative-ion modes were used. A total of 100 compounds were putatively annotated based on accurate mass, retention time, MS/MS fragmentation matching, and fragmentation scores using the LuMet-TCM database (Table S2).

**Figure 6:**
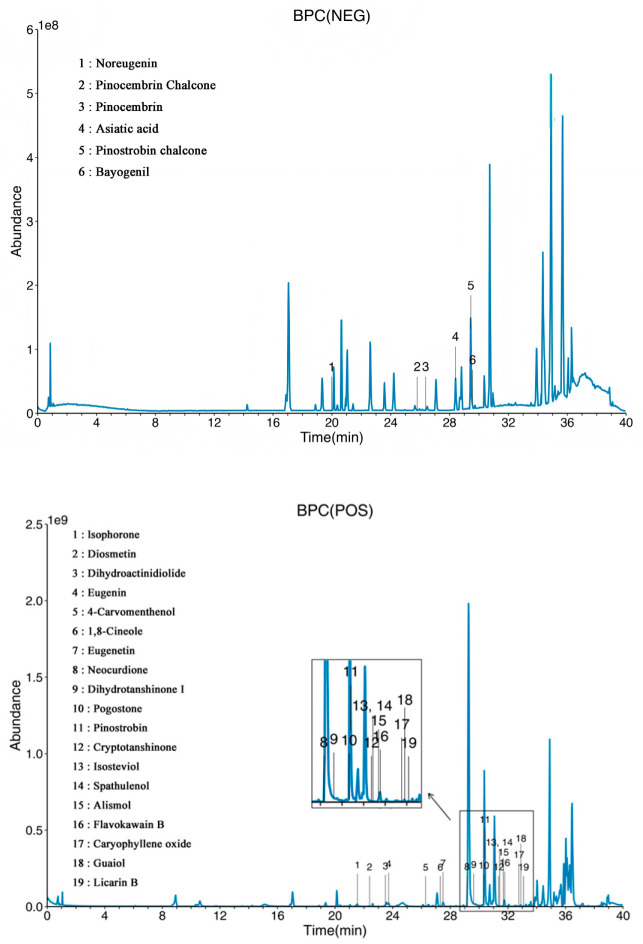
Base peak chromatograms (BPC) of the ethanolic extract of BF in positive- and negative-ion modes.

Among these putatively annotated compounds, 25 BF-derived candidate metabolites were selected for further physicochemical and pharmacokinetic evaluation (Figure 6 and Table 1). The retained compounds mainly belonged to plant secondary metabolites, including terpenoids, flavonoids, chalcones, phenylpropanoids, and triterpenoids. Based on drug-likeness and toxicity prediction, 17 compounds were retained for subsequent network pharmacology analysis.

This screening process provided a focused compound set for exploring the potential pharmacological basis of BF. Nevertheless, because the UHPLC-Q Exactive Orbitrap-HRMS-based annotations were putative and quantitative analysis was not performed, the abundance and individual contribution of these metabolites remain unclear. Therefore, the network pharmacology results should be interpreted only as hypothesis-generating evidence rather than direct confirmation of the active constituents of BF.

### 2.7. Network Pharmacology Analysis

**Figure 7:**
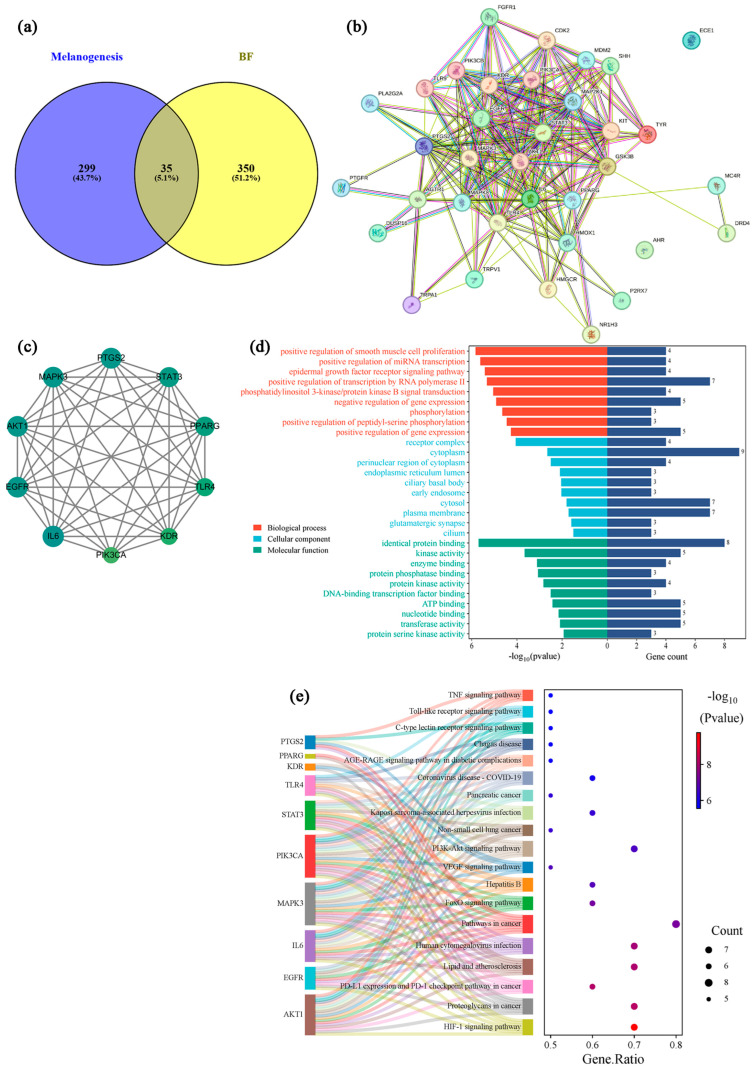
PPI network and hub gene analysis. (**a**) Overlap between targets of BF-derived candidate compounds and melanogenesis-related targets; (**b**) PPI network of potential melanogenesis-related targets; (**c**) Top 10 hub genes ranked by maximal clique centrality (MCC) scores. Node color intensity represents the relative MCC score, with darker colors indicating higher scores; (**d**) GO enrichment analysis. The left bar plot shows the significance level expressed as −log_10_(*p* value), and the right bar plot shows the gene count for each GO term; (**e**) KEGG pathway enrichment analysis. Bubble size represents gene count, and color indicates enrichment significance (−log_10_(*p* value)).

To identify potential interactions between compounds and melanogenesis-related targets, a two-step approach was employed. First, the web SwissTargetPrediction was used to predict 334 target genes associated with 17 compounds. Second, melanogenesis-related genes were retrieved from the GeneCards website, yielding 385 potential target genes. Comparative analysis of these datasets identified 35 overlapping target genes (Figure 7a). A total of 35 overlapping targets were used to construct a protein–protein interaction (PPI) network (Figure 7b). Hub gene analysis identified 10 key targets, including IL6, MAPK3, EGFR, PTGS2, AKT1, STAT3, PPARG, TLR4, KDR, and PIK3CA (Figure 7c). These shared targets may represent candidate targets of the antimelanogenic effects, as they are both predicted targets of the candidate metabolites and implicated in melanogenesis-related processes.

To explore the biological significance of candidate metabolites of BF in melanogenesis, Gene Ontology (GO) enrichment analysis was performed on 35 candidate targets using the DAVID database. A total of 155 GO terms were identified, including 100 biological processes (BP), 18 cellular components (CC), and 37 molecular functions (MF) (Figure 7d).

In BP terms, the enriched terms were mainly associated with phosphorylation, positive regulation of gene expression, and phosphatidylinositol 3-kinase/AKT signaling pathway. In addition, processes such as epidermal growth factor receptor signaling and regulation of transcription were also observed. These findings may indicate that BF-related targets participate in intracellular signaling cascades and transcriptional regulation, which have been reported to be involved in melanogenesis and cellular stress responses.

For CC analysis, the targets were primarily enriched in the plasma membrane, cytoplasm, cytosol, endoplasmic reticulum lumen, and early endosome. These subcellular localizations suggest that the predicted targets may be distributed across multiple cellular compartments, ranging from membrane-associated receptors to intracellular signaling and protein processing sites. Considering that melanogenesis involves receptor activation, signal transduction, and melanosome formation, the observed distribution may reflect a coordinated regulatory process within different cellular regions.

Regarding MF, the enriched terms mainly included kinase activity, protein serine kinase activity, DNA-binding transcription factor binding, and protein phosphatase binding. These functions are generally associated with phosphorylation-dependent signaling and transcriptional regulation. BF may influence key regulatory proteins involved in melanogenesis, such as MITF, through modulation of kinase activity and downstream gene expression.

KEGG enrichment analysis indicated that pathways such as PI3K–Akt signaling may be involved in the biological effects of BF (Figure 7e). The PI3K–Akt pathway is known to regulate melanogenesis through modulation of MITF and downstream melanogenic enzymes [35], which is consistent with our experimental findings.

Overall, the GO and KEGG enrichment analyses suggested that BF may exert its biological effects through coordinated regulation of phosphorylation-related signaling pathways, transcriptional activity, and multi-compartment cellular processes. These findings provide a basis for further investigation into the potential mechanisms underlying the antimelanogenic of BF.

### 2.8. Molecular Docking Analysis

Molecular docking was performed as an in silico, hypothesis-generating approach to evaluate potential interactions between metabolites and key proteins involved in melanogenesis. The analysis estimated binding conformations and relative binding energies, providing supportive but not definitive evidence of compound-target interactions. Lower binding energies were interpreted as indicating a more stable predicted compound-protein binding [36,37].

**Figure 8:**
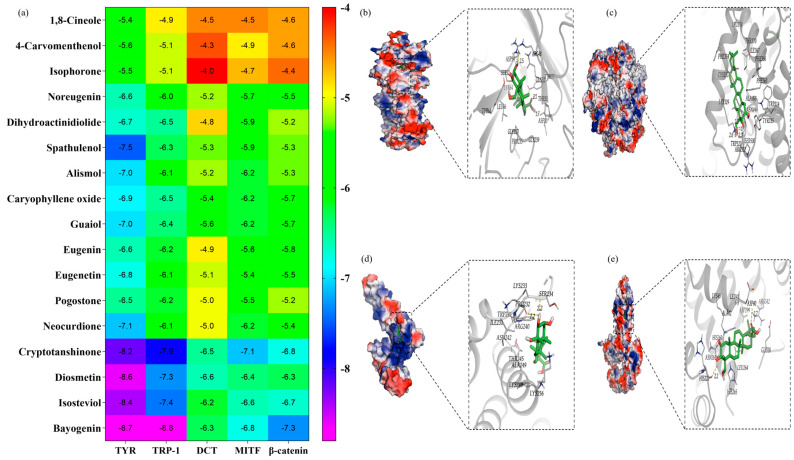
(**a**) Predicted binding energies of 17 metabolites with TYR, TRP-1, DCT, MITF and β-catenin obtained from molecular docking analysis. (**b**–**e**) Predicted binding modes of bayogenin with TYR, TRP-1, MITF, and β-catenin, respectively.

Based on the observed changes in total protein expression, TYR, TRP-1, DCT, MITF, and β-catenin were selected as candidate targets for subsequent molecular docking analysis. The selected compounds were docked against these representative melanogenesis-related targets (Figure 8a). Overall, most compounds exhibited moderate to strong binding affinities, with binding energies ranging from −4.0 to −8.8 kcal/mol. Among them, cryptotanshinone, diosmetin, and bayogenin showed relatively low binding energies toward multiple targets, with bayogenin demonstrating comparatively stronger predicted binding.

Further analysis suggested that bayogenin could be accommodated within the binding pockets of several targets (Figure 8b–e). In TYR, bayogenin was predicted to form hydrogen bonds with residues Asp30, Thr31, and Asp54, which may contribute to stabilizing the ligand–protein complex and potentially influence enzyme activity. In TRP-1, hydrogen bond interactions were predicted with Ala150 and Gln155, suggesting a relatively stable binding conformation. In MITF, bayogenin was predicted to interact with residues Ser234 and Pro232, while in β-catenin, interactions were identified with His223 and Asp40. These interactions may contribute to the stabilization of ligand–protein complexes and suggest potential binding affinity toward these targets.

The docking results provide supportive, hypothesis-generating insights rather than definitive evidence of direct protein–ligand interactions or functional modulation. The role of bayogenin in melanogenesis remains largely unclear, and there is currently limited direct evidence linking it to melanogenesis-related signaling pathways. Therefore, these findings should be interpreted with caution and regarded as preliminary, hypothesis-generating evidence for prioritizing candidate metabolites for further experimental validation.

## 3. Discussion

This study employed a series of experiments to evaluate the skin-whitening potential of *Baeckea frutescens* (BF), providing experimental evidence for its antimelanogenic activity. Within non-cytotoxic concentration ranges, BF reduced intracellular tyrosinase activity and melanin content in α-MSH-stimulated B16F10 cells, along with decreased expression of MITF and its downstream melanogenic enzymes (TYR, TRP-1, and DCT).

To further explore the underlying mechanisms, the involvement of PKA/CREB, AKT, β-catenin, and ERK/MAPK pathways in MITF regulation was investigated. The results showed that BF reduced the phosphorylation levels of PKA, CREB and ERK, while decreasing the total and phosphorylated AKT and β-catenin levels. These signaling pathways play important roles in melanogenesis and operate at multiple regulatory levels [31,38]. The cAMP/PKA/CREB axis directly regulates MITF transcription, while ERK signaling can influence MITF stability and degradation [31]. In parallel, β-catenin signaling contributes to transcriptional activation of MITF, while AKT signaling may modulate melanogenesis partly through its influence on β-catenin-related mechanisms [33].

The concurrent modulation of these pathways by BF suggests a coordinated regulatory effect on MITF expression rather than the involvement of a single signaling cascade. Although p-GSK-3β (Ser9) was increased, β-catenin was decreased. This observation differs from the classical interpretation that Ser9 phosphorylation stabilizes β-catenin, indicating that its regulation is not governed by a single signaling axis. In addition, β-catenin stability is regulated by multiple phosphorylation sites and signaling inputs, and therefore changes in a single regulatory axis may not fully determine its overall protein level [33]. This difference may be due to the complex nature of signaling regulation, especially when using plant extracts that contain multiple bioactive components acting on different targets.

The in vivo zebrafish model further supported these findings, as BF treatment was associated with reduced melanin accumulation. As the extract was evaluated as a whole system, the observed biological effects may result from the combined action of multiple constituents. Therefore, the contributions of individual compounds and their potential synergistic interactions could not be specifically determined under the current experimental conditions.

Taken together, these findings suggest that BF possesses antimelanogenic potential and could serve as a promising candidate for skin-whitening applications. Further studies are required to validate its efficacy and safety in mammalian models and clinical settings, as well as to clarify the precise molecular mechanisms underlying its activity.

## 4. Materials and Methods

### 4.1. Materials

Mushroom tyrosinase, α-arbutin, α-MSH and 3,4-dihydroxyphenylalanine (L-DOPA) were purchased from Sigma-Aldrich (St. Louis, MO, USA). Primary antibodies against TYR, DCT, MITF, CREB, p-CREB (S133), PKA C-α, p-PKA C-α, AKT, p-AKT (S473), ERK1/2, p-ERK1/2 (T202/Y204), JNK 1/2/3, p-JNK 1/2/3 (T183/T183/T221), GSK-3β, p-GSK-3β (S9), β-catenin, and β-actin were purchased from ABclonal (Woburn, MA, USA). TRP-1 was purchased from Abcam (Cambridge, UK). p-β-catenin (S675) was obtained from Cell Signaling Technology (Danvers, MA, USA). RIPA lysis buffer (Strong) was purchased from Beyotime Biotechnology (Jiangsu, China). B16F10 cells were obtained from BeNa Culture Collection (Xinyang, China). Unless otherwise stated, all other chemicals were purchased from Sigma-Aldrich.

### 4.2. Preparation of Ethanol Extract from Baeckea frutescens L.

Wild *Baeckea frutescens* L. was purchased from Dashan Buluo Wild Herbal Medicine (Qinzhou, Guangxi, China). The plant material consisted of shade-dried leaves and stems. The botanical identity was authenticated by Hongjuan Bao (Xiamen Medical College, Xiamen, China), and a voucher specimen was deposited at the School of Pharmacy, Xiamen Medical College. The shade-dried leaves and stems of BF were ground into fine powder. The powder was extracted with ethanol at a material-to-solvent ratio of 1:10 (*w*/*v*) and soaked at 4 °C for 12 h, and subsequently subjected to ultrasonic-assisted extraction using an ultrasonic cleaner (KQ-500DE, Kunshan Ultrasonic Instruments Co., Kunshan, China) at 40 kHz and 500 W for 30 min at room temperature. The extract was filtered, concentrated under reduced pressure, and freeze-dried to obtain the ethanol extract. The extract was stored or diluted to the desired concentration with ethanol at −20 °C for later use.

### 4.3. Mushroom Tyrosinase Inhibitory Capacity Assay

The residue was diluted in ethanol to prepare samples of different concentrations. The 3 groups were set up: experimental group (EG), background group (BG), and control group (CG). Samples (200 µL) were added to EG and BG, while the same volume of ethanol was added to CG. L-DOPA (300 µL, 5 mM) was added to all groups and mixed thoroughly, and then pre-incubated at 37 °C for 10 min. Mushroom tyrosinase solution (100 µL, 400 U/mL) was added to EG and CG; an equivalent volume of PBS (pH 7.4) was added to BG. After mixing, the reaction solution was incubated in the dark for 20 min, and the absorbance at 475 nm (A_475_) was recorded. Ascorbic acid was used as a reference compound in the mushroom tyrosinase inhibition assay. The IC_50_ values of BF and ascrobic acid were calculated from concentration-response curves by nonlinear regression. The tyrosinase inhibition rate was calculated according to Formula (1).

(1) $$Mushroom\;Tyrosinase\;Inhibition=[1-(A_{EG}-A_{BG})/A_{CG}]\times 100\%$$

### 4.4. Cell Culture

B16F10 cells were cultured in Dulbecco’s Modified Eagle Medium (DMEM) supplemented with 10% fetal bovine serum and 1% penicillin-streptomycin and maintained at 37 °C in a humidified incubator containing 5% CO_2_.

### 4.5. Cell Viability Assay

B16F10 cell viability was evaluated using the MTT assay (3-(4,5-dimethylthiazol-2-yl)-2,5-diphenyltetrazolium bromide). Cells were seeded at a density of 1 × 10^5^ cells/mL in 96-well plates and incubated overnight. The medium (100 μL) was replaced with fresh medium containing samples at different concentrations (0–200 μg/mL) and incubated for 24 h. MTT solution (10 μL, 5 mg/mL) was added to each well, followed by incubation at 37 °C for 4 h. The supernatant was removed, and 150 μL of dimethyl sulfoxide (DMSO) was added to solubilize the resulting formazan crystals. After shaking for 10 min, the absorbance was measured at 570 nm.

### 4.6. Melanin Content Assay

B16F10 cells at approximately 80% confluence were seeded into 6-well plates and incubated overnight. The four groups were assigned: control, α-MSH, α-arbutin (positive control), and sample groups. The next day, the α-arbutin group received 0.5 mM α-arbutin, the sample groups received various concentrations of BF, and 10 μM α-MSH was added to the α-MSH, α-arbutin, and sample groups; the control group was brought to the same final volume with medium. α-Arbutin was used as a reference compound to confirm the responsiveness of the α-MSH-induced B16F10 melanogenesis model, rather than as a direct molar-equivalent comparator for BF. After 24 h, cells were lysed in 200 μL of 0.5% Triton X-100 for 10 min at 4 °C with gentle shaking, followed by sonication for 30 s using a pulse mode (2 s on, 1 s off). The lysates were centrifuged at 12,000× *g* for 10 min. The supernatant was collected for intracellular tyrosinase activity assays, while the residue was used for melanin content determination. For melanin content analysis, the residue was dissolved in 100 μL of 1 M NaOH containing 10% DMSO and heated at 100 °C for 10 min. After cooling to room temperature, the absorbance was measured at 475 nm.

### 4.7. Intracellular Tyrosinase Activity Assay

The supernatant (50 μL) was combined with 150 μL of 10 mM L-Dopa, gently mixed, and incubated at 37 °C for 30 min. The absorbance was measured at 475 nm.

### 4.8. Western Blot

Following the preceding treatments, B16F10 cells were lysed on ice for 30 min in RIPA buffer supplemented with 1 mM PMSF. Lysates were transferred to 1.5 mL microtubes and centrifuged at 12,000× *g* for 10 min at 4 °C. The supernatant was collected and mixed with loading buffer, followed by denaturation at 100 °C for 5 min. Protein concentrations were determined using a BCA assay and adjusted to equal levels. Equal amounts of protein (20 μg) were separated by SDS-PAGE and subsequently transferred onto PVDF membranes. The membranes were blocked with 5% bovine serum albumin at room temperature for 1 h, incubated with primary antibodies (1:3000) overnight at 4 °C with gentle shaking. After three washes with TBST (5 min each), the membranes were incubated with HRP-conjugated secondary antibodies (1:5000) for 1 h at room temperature. After three washes with TBST (5 min each), protein bands were visualized using NcmECL Ultra reagent (NCM Biotech, Suzhou, China) and imaged using a ChemiDoc^TM^ XRS+ Imaging System (Bio-Rad, Hercules, CA, USA).

### 4.9. Determination of Zebrafish Embryo Mortality Rate

Zebrafish embryos were collected 6 h post-fertilization and placed in culture wells. They were treated with BF at final concentrations of 0, 12.5, 25, 50, 100, and 200 μg/mL at final concentrations. Each sample group contained 30 zebrafish embryos. The embryos were incubated in a constant-temperature incubator at 28 °C for 72 h, with an adjusted light/dark ratio of 14 h:10 h [39]. The hatching rate and survival status of the embryos were recorded. All animal experimental procedures were approved by the Medical Ethics Committee of Xiamen Medical College (approval code: 20240306014, Xiamen, China).

### 4.10. Evaluation of the Antimelanogenic Effect on Zebrafish Embryos

Zebrafish melanogenesis assays were performed by Hunter Biotech (Hangzhou, China). After assessing survival rates, the antimelanogenic effects were further evaluated in zebrafish embryos using non-toxic concentrations of BF. Thirty embryos within 6 h post-fertilization were placed in culture wells and treated with samples at different final concentrations of 12.5, 25, and 50 μg/mL, using α-arbutin as the positive control. The incubation was carried out at 28 °C for 48 h. Ten surviving zebrafish larvae were randomly selected from each experimental group using a stereomicroscope (Olympus SZX7, Hachioji-shi, Japan) to capture digital images. Melanin signal intensity in the zebrafish larvae was analyzed using ImageJ software (version 1.53k; NIH, Bethesda, MD, USA).

### 4.11. UHPLC–Q Exactive Orbitrap-HRMS Analysis and Compound Annotation

The same freeze-dried BF ethanol extract used for the biological assays was subjected to chemical profiling to ensure consistency between the chemical and biological evaluations. Briefly, 50 mg of the BF extract was reconstituted in 300 μL of ethanol, transferred into a 1.5 mL centrifuge tube, vortexed for 1 min, and sonicated in an ice-water bath for 30 min to ensure complete dissolution and homogenization. This sonication step was performed to facilitate sample dissolution rather than as a secondary extraction procedure. After centrifugation at 12,000× *g* for 10 min at 4 °C, 190 μL of the supernatant was transferred to an LC–MS vial, and 10 μL of internal standard was added prior to analysis.

Metabolite profiling was performed using an ultra-high-performance liquid chromatography system coupled with a Q Exactive Orbitrap high-resolution mass spectrometer (UHPLC-Q Exactive Orbitrap-HRMS) at OE Biotech Co., Ltd. (Shanghai, China). Chromatographic separation was achieved using an ACQUITY UPLC I-Class system (Waters Corporation, Milford, MA, USA) coupled with a Q Exactive HF Orbitrap mass spectrometer (Thermo Fisher Scientific, Waltham, MA, USA), equipped with an ACQUITY UPLC HSS T3 column (100 mm × 2.1 mm, 1.8 μm).

Mass spectrometry data were acquired in both positive- and negative-ion modes. Detailed chromatographic gradients and MS parameters are provided in Tables S3–S5. Raw data were processed using XCMS (version 4.5.1) for peak detection, alignment, and retention time correction.

Compound annotation was performed using the LuMet-TCM database, which was established based on reference standard information under comparable LC–MS conditions. The putative annotations were assigned according to adduct information, retention time, accurate mass matching between theoretical and observed m/z values, mass error, MS/MS spectral matching, with retention time deviation ≤ 0.3 min and mass error ≤ 5 ppm used as annotation criteria. For compounds with acquired MS/MS spectra, secondary fragmentation information was used to support annotation, and major fragment ions and fragmentation scores are provided in Table S2 when available. Since authentic reference standards were not individually analyzed for all annotated compounds, these assignments were regarded as putative annotations. In addition, because systematic literature-based verification of whether each compound has been previously reported in *B. frutescens* was beyond the scope of this study, no definitive claim is made regarding newly identified constituents.

### 4.12. Network Pharmacological Analysis of BF

The compounds putatively annotated by UHPLC-Q Exactive Orbitrap-HRMS were further screened to obtain candidate BF-derived metabolites for network pharmacology analysis. Common endogenous small molecules, including amino acids, nucleotides, low-molecular-weight carbohydrates, and lipids, were excluded. SwissADME (http://www.swissadme.ch/, accessed on 14 January 2026) was used to evaluate physicochemical properties and drug-likeness, mainly according to Lipinski’s Rule of Five. The screening criteria included MW ≤ 500 Da, nRotB ≤ 10, nHBA ≤ 10, nHBD ≤ 5, TPSA < 140 Å^2^, LogP < 5, and LogKp ≥ −6 cm/s. Toxicity-related properties were predicted using pkCSM (https://biosig.lab.uq.edu.au/pkcsm/, accessed on 14 January 2026). Compounds predicted to have potential AMES toxicity or hepatotoxicity were excluded from further analysis.

It should be noted that the selected metabolites were not confirmed by authentic standards, and their concentrations in the BF extract were not determined. Therefore, the network pharmacology analysis was performed as an exploratory and hypothesis-generating approach, rather than as definitive evidence that these metabolites are responsible for the biological effects of BF.

The simplified molecular input line entry system (SMILES) structures of the candidate compounds from *Baeckea frutescens* (BF) were obtained from the PubChem database (https://pubchem.ncbi.nlm.nih.gov/, accessed on 14 January 2026). These SMILES data were subsequently uploaded to the SwissTargetPrediction platform (https://www.swisstargetprediction.ch/, accessed on 14 January 2026) to predict potential protein targets. Redundant targets were removed to ensure data accuracy.

Skin pigmentation-related targets were collected by querying the keywords “Melanogenesis” in databases, including GeneCards (https://www.genecards.org/, accessed on 14 January 2026, relevance score > 0.5) and OMIM (https://www.omim.org/, accessed on 14 January 2026). After merging and deduplication, the overlapping targets between BF-derived candidate compounds and pigmentation-related genes were identified using Venn analysis via the VENNY 2.1.0 online tool (https://bioinfogp.cnb.csic.es/tools/venny/index.html, accessed on 16 January 2026).

To elucidate the molecular mechanism of BF in melanogenesis regulation, intersecting targets were imported into the STRING database (https://string-db.org/, accessed on 14 January 2026) to construct a PPI network. The species was set to *Homo sapiens*, and interactions with a confidence score > 0.4 were retained. The interaction data were further analyzed using Cytoscape (version 3.8.0; Cytoscape Consortium, San Diego, CA, USA), and hub genes were identified using the CytoHubba plugin based on MCC scores [40].

Functional enrichment analyses, including Gene Ontology (GO) and Kyoto Encyclopedia of Genes and Genomes (KEGG) pathway analyses, were conducted using the DAVID database (https://davidbioinformatics.nih.gov, accessed on 18 January 2026). GO terms were categorized into biological process (BP), molecular function (MF), and cellular component (CC). KEGG pathways and GO terms with *p* < 0.05 were considered statistically significant. The top 10 enriched GO terms and 19 KEGG pathways were selected for visualization to elucidate the potential mechanisms of BF in regulating melanogenesis.

Finally, a network integrating compounds, targets, and pathways was constructed using Cytoscape, based on the top 15 KEGG pathways and the PPI network, providing a preliminary overview of the multi-component, multi-target, and multi-pathway mechanisms.

### 4.13. Molecular Docking

To further explore the potential interactions predicted by network pharmacology, molecular docking was performed between key metabolites and core target proteins, including TYR, TRP-1, DCT, MITF, and β-catenin.

The crystal structures of TYR (PDB ID: 7RK7), TRP-1 (PDB ID: 8WPL), DCT (PDB ID: 4HX1), MITF (PDB ID: 7EOD), and β-catenin (PDB ID: 1G3J) were obtained from the RCSB Protein Data Bank (https://www.rcsb.org/, accessed on 17 January 2026). Protein structures were preprocessed using PyMOL 3.1 (http://www.pymol.org/, accessed on 17 January 2026) by removing water molecules and phosphate ions. Hydrogen atoms were added, and docking grids were defined based on the active binding sites.

All protein structures were converted from PDB format to PDBQT format using AutoDockTools (Version 1.5.7; The Scripps Research Institute, La Jolla, CA, USA). Ligand structures were energy-minimized and also converted into PDBQT format prior to docking. Molecular docking simulations were conducted using AutoDock Vina (Version 1.1.2). The binding conformation with the lowest calculated binding energy (kcal/mol) was selected as the optimal mode. Lower binding energy values were interpreted as indicative of more stable ligand–target interactions.

In this study, molecular docking was employed as a hypothesis-generating approach to explore potential ligand–target interactions. The docking parameters were set based on established protocols. Although redocking and RMSD-based validation were not explicitly performed, the predicted binding modes were evaluated by analyzing key interactions within the active sites and were found to be consistent with previously reported ligand–protein interaction patterns. Therefore, the docking results were interpreted with caution and used as supportive evidence.

### 4.14. Statistical Analysis

The experimental results were analyzed and visualized using SigmaPlot software (Version 14.0; Systat Software Inc., San Jose, CA, USA). Statistical analyses were performed using one-way analysis of variance (ANOVA). Homogeneity of variances was assessed using the built-in tests in SigmaPlot software. Depending on the results, Tukey’s test was applied when equal variances were assumed, while Dunnett’s T3 test was used when variance heterogeneity was present. Holm–Sidak’s test was used for specific pairwise comparisons where appropriate. Data are presented as mean ± standard deviation (SD), and a *p*-value < 0.05 was considered statistically significant.

## 5. Conclusions

In conclusion, BF exhibited antimelanogenic activity by reducing melanogenesis, inhibiting tyrosinase activity, and downregulating MITF and its downstream enzymes. Mechanistically, these effects may be associated with coordinated modulation of PKA/CREB, ERK/MAPK, and β-catenin signaling pathways. The combined in vitro, in vivo, and in silico findings suggest that BF may serve as a promising candidate for further development as a natural skin-whitening agent. Nevertheless, further studies are required to identify its candidate metabolites and validate its efficacy and safety in clinical settings.

## Figures and Tables

**Table 1:** Physicochemical and pharmacokinetic properties of selected BF-derived candidate metabolites predicted by SwissADEM and pkCSM servers.

No.	Compounds	Physicochemical Properties							Pharmacokinetic Properties				
		MW (g/mol)	nRotB	nHBA	nHBD	TPSA (Å^2^)	logP	Log Kp (cm/s)	AMES Toxicity	hERG I Inhibitor	hERG II Inhibitor	Hepato- toxicity	Skin Sen- sitisation
1	1,8-Cineole	154.25	1	1	0	9.23	2.67	−5.3	No	No	No	No	Yes
2	4-Carvomenthenol	154.25	1	1	1	20.23	2.6	−4.93	No	No	No	No	Yes
3	Isophorone	138.21	1	1	0	17.07	2.11	−5.94	No	No	No	No	Yes
4	Noreugenin	192.17	4	4	2	70.67	1.49	−5.87	No	No	No	No	No
5	Dihydroactinidiolide	180.24	2	2	0	26.3	2.41	−5.87	No	No	No	No	Yes
6	Pinocembrin Chalcone	256.25	4	4	3	77.76	2.18	−5.61	Yes	No	No	No	No
7	Pinocembrin	256.25	4	4	2	66.76	2.26	−5.82	Yes	No	No	No	No
8	Pinostrobin chalcone	270.28	4	4	2	66.76	2.62	−5.46	Yes	No	Yes	No	No
9	Spathulenol	220.35	1	1	1	20.23	3.3	−5.44	No	No	No	No	Yes
10	Alismol	220.35	1	1	1	20.23	3.3	−5.73	No	No	No	No	Yes
11	Caryophyllene oxide	220.35	1	1	0	12.53	3.68	−5.12	No	No	No	No	Yes
12	Guaiol	222.37	1	1	1	20.23	3.42	−5.48	No	No	No	No	Yes
13	Eugenin	206.19	4	4	1	59.67	1.89	−5.73	No	No	No	No	No
14	Eugenetin	220.22	4	4	1	59.67	2.12	−5.9	No	No	No	No	No
15	Pogostone	224.25	4	4	1	67.51	2.15	−5.83	No	No	No	No	No
16	Neocurdione	236.35	2	2	0	34.14	3	−5.86	No	No	No	No	Yes
17	Pinostrobin	270.28	4	4	1	55.76	2.66	−5.68	Yes	No	No	No	No
18	Flavokawain B	284.31	4	4	1	55.76	3.06	−5.31	Yes	No	Yes	Yes	No
19	Cryptotanshinone	296.36	3	3	0	43.37	3.43	−5.41	No	No	No	No	No
20	Diosmetin	300.26	6	6	3	100.13	2.19	−5.93	No	No	No	No	No
21	Dihydrotanshinone I	278.3	3	3	0	43.37	3.03	−5.75	No	No	No	Yes	No
22	Isosteviol	318.45	3	3	1	54.37	3.84	−5.15	No	No	No	No	No
23	Licarin B	324.37	4	4	0	36.92	4.11	−5.03	No	No	No	Yes	No
24	Asiatic acid	488.7	5	5	4	97.99	4.45	−5.23	No	No	No	Yes	No
25	Bayogenin	488.7	5	5	4	97.99	4.55	−5.13	No	No	No	No	No

## Data Availability

The original contributions presented in this study are included in the article/Supplementary Material. Further inquiries can be directed to the corresponding authors.

## Supplementary Materials

The following supporting information can be downloaded at: https://www.mdpi.com/article/10.3390/molecules31101685/s1, Figure S1: Effect of BF on JNK 1/2/3 MAPK signaling in B16F10 cells; Table S1: Number of deaths and mortality rates of Zebrafish embryos (*n* = 30); Table S2: UHPLC-Q Exactive Orbitrap-HRMS-based putative annotation of metabolites in the ethanolic extract of *B. frutescens* under positive- and negative-ion modes [41,42,43,44]; Table S3: The parameters of the heated electrospray ionization-mass spectrometry (HESI-MS) of positive- and negative-ion models; Table S4: Instrument operation program of LC-MS analysis; Table S5: Changes in solvents in gradient elution of LC-MS analysis.

## References

[1] Dobosz M., Radziwon J., Cubała W.J. (2024). Worldwide internet trends in the public interest related to skin whitening and bleaching creams. J. Cosmet. Laser Ther..

[2] Masub N., Khachemoune A. (2022). Cosmetic skin lightening use and side effects. J. Dermatol. Treat..

[3] Zhao W., Yang A., Wang J., Huang D., Deng Y., Zhang X., Qu Q., Ma W., Xiong R., Zhu M. (2022). Potential application of natural bioactive compounds as skin-whitening agents: A review. J. Cosmet. Dermatol..

[4] Leong H.J.-Y., Teoh M.-L., Beardall J., Convey P. (2024). Green beauty unveiled: Exploring the potential of microalgae for skin whitening, photoprotection and anti-aging applications in cosmetics. J. Appl. Phycol..

[5] Peng X., Ma Y., Yan C., Wei X., Zhang L., Jiang H., Ma Y., Zhang S., Xing M., Gao Y. (2024). Mechanism, formulation, and efficacy evaluation of natural products for skin pigmentation treatment. Pharmaceutics.

[6] Pillaiyar T., Namasivayam V., Manickam M., Jung S.-H. (2018). Inhibitors of melanogenesis: An updated review. J. Med. Chem..

[7] Abdel-Hakim A., El-Maghrabey M., Tsubokami A., Belal F., Hammad M.A., Kuroda N., Kishikawa N. (2026). Applying pulse UV irradiation-induced chemiluminescence approach for high-throughput screening assay of tyrosinase inhibitors. Talanta.

[8] Khan M., Xin J., Ahmed I., Zhang T., Feng X., Gao P., Li J. (2025). Drug repurposing and AI-driven discovery of tyrosinase inhibitors, emerging strategies for skin disorders: A review. Int. J. Biol. Macromol..

[9] D’Mello S.A., Finlay G.J., Baguley B.C., Askarian-Amiri M.E. (2016). Signaling pathways in melanogenesis. Int. J. Mol. Sci..

[10] LEE T.H., LEE M.S. (1971). Studies on MSH-induced melanogenesis: Effect of long-term administration of MSH on the melanin content and tyrosinase activity. Endocrinology.

[11] Seo S.-H., Jo J.K., Kim E.-J., Park S.-E., Shin S.Y., Park K.M., Son H.-S. (2020). Metabolomics reveals the alteration of metabolic pathway by alpha-melanocyte-stimulating hormone in B16F10 melanoma cells. Molecules.

[12] Vachtenheim J., Borovansky J. (2010). “Transcription physiology” of pigment formation in melanocytes: Central role of MITF. Exp. Dermatol..

[13] Dall’Olmo L., Papa N., Surdo N.C., Marigo I., Mocellin S. (2023). Alpha-melanocyte stimulating hormone (alpha-MSH): Biology, clinical relevance and implication in melanoma. J. Transl. Med..

[14] Matsuzawa A., Peng H.-Y., Lin C.-C., Wang H.-Y., Shih Y., Chou S.-T. (2014). The Melanogenesis Alteration Effects of Achillea millefolium L. Essential Oil and Linalyl Acetate: Involvement of Oxidative Stress and the JNK and ERK Signaling Pathways in Melanoma Cells. PLoS ONE.

[15] Herraiz C., Journe F., Abdel-Malek Z., Ghanem G., Jimenez-Cervantes C., García-Borrón J.C. (2011). Signaling from the human melanocortin 1 receptor to ERK1 and ERK2 mitogen-activated protein kinases involves transactivation of cKIT. Mol. Endocrinol..

[16] Bellei B., Flori E., Izzo E., Maresca V., Picardo M. (2008). GSK3β inhibition promotes melanogenesis in mouse B16 melanoma cells and normal human melanocytes. Cell Signal..

[17] Ferreira A.M., de Souza A.A., Koga R.C.R., Sena I.D.S., Matos M.J.S., Tomazi R., Ferreira I.M., Carvalho J.C.T. (2023). Anti-Melanogenic Potential of Natural and Synthetic Substances: Application in Zebrafish Model. Molecules.

[18] Meng Y., Zhou J.-X., Yang Y.-T., Wei X.-P., Li S.-Y., Ni H.-G. (2025). Unsupervised SAM segmentation of zebrafish body: Application to melanin analysis. Environ. Pollut..

[19] Ma Y.-M., Zhang X.-Z., Su Z.-Z., Li N., Cao L., Ding G., Wang Z.-Z., Xiao W. (2015). Insight into the molecular mechanism of a herbal injection by integrating network pharmacology and in vitro. J. Ethnopharmacol..

[20] Li S., Zhang B. (2013). Traditional Chinese medicine network pharmacology: Theory, methodology and application. Chin. J. Nat. Med..

[21] Zhao N., Kong H., Liu H., Shi Q., Qi X., Chen Q. (2022). A network pharmacology approach to evaluate the synergistic effect of dihydromyricetin and myricitrin in vine tea on the proliferation of B16F10 cells. Front. Nutr..

[22] Yu X., Wang Y., Wu Z., Jia M., Xu Y., Qu H., Zhao X., Wang S., Jing L., Lou Y. (2024). Multi-technology integrated network pharmacology-based study on phytochemicals, active metabolites, and molecular mechanism of Psoraleae Fructus to promote melanogenesis. J. Ethnopharmacol..

[23] Kamiya K., Satake T. (2010). Chemical constituents of Baeckea frutescens leaves inhibit copper-induced low-density lipoprotein oxidation. Fitoterapia.

[24] Kamarazaman I.S., Kiong L.S., Hasan M.K.N., Basherudin N., Kasim N.A.M., Ali A.A., Ramli S., Maniam S., James R.J., Rojsitthisak P. (2024). Baeckea frutescens L. Promotes wound healing by upregulating expression of TGF-β, IL-1 β, VEGF and MMP-2. Saudi Pharm. J..

[25] Nisa K., Nurhayati S., Apriyana W., Indrianingsih A. (2017). Investigation of total phenolic and flavonoid contents, and evaluation of antimicrobial and antioxidant activities from Baeckea frutescens extracts. Proceedings of the IOP Conference Series: Earth and Environmental Science.

[26] Park K.M., Kwon K.M., Lee S.H. (2015). Evaluation of the Antioxidant Activities and Tyrosinase Inhibitory Property from Mycelium Culture Extracts. Evid. Based Complement. Altern. Med..

[27] Ghani U. (2022). Azole inhibitors of mushroom and human tyrosinases: Current advances and prospects of drug development for melanogenic dermatological disorders. Eur. J. Med. Chem..

[28] Wen S.Y., Wu Y.S., Liu H., Ng S.C., Padma V.V., Huang C.Y., Kuo W.W. (2023). Paeoniflorin found in Paeonia lactiflora root extract inhibits melanogenesis by regulating melanin-related signal transduction in B16F10 cells. J. Cosmet. Dermatol..

[29] Liu J., Xu X., Zhou J., Sun G., Li Z., Zhai L., Wang J., Ma R., Zhao D., Jiang R. (2023). Phenolic acids in Panax ginseng inhibit melanin production through bidirectional regulation of melanin synthase transcription via different signaling pathways. J. Ginseng Res..

[30] Park S., Han N., Lee J., Lee J.N., An S., Bae S. (2023). Anti-Melanogenic Effects of Lilium lancifolium Root Extract via Downregulation of PKA/CREB and MAPK/CREB Signaling Pathways in B16F10 Cells. Plants.

[31] Nam G., An S.K., Park I.C., Bae S., Lee J.H. (2022). Daphnetin inhibits alpha-MSH-induced melanogenesis via PKA and ERK signaling pathways in B16F10 melanoma cells. Biosci. Biotechnol. Biochem..

[32] Hong C., Zhang Y., Yang L., Xu H., Cheng K., Lv Z., Chen K., Li Y., Wu H. (2024). Epimedin B exhibits pigmentation by increasing tyrosinase family proteins expression, activity, and stability. J. Pharm. Anal..

[33] Liu J., Xiao Q., Xiao J., Niu C., Li Y., Zhang X., Zhou Z., Shu G., Yin G. (2022). Wnt/β-catenin signalling: Function, biological mechanisms, and therapeutic opportunities. Signal Transduct. Target. Ther..

[34] Beurel E., Grieco S.F., Jope R.S. (2015). Glycogen synthase kinase-3 (GSK3): Regulation, actions, and diseases. Pharmacol. Ther..

[35] Pang M., Xu R., Xi R., Yao H., Bao K., Peng R., Zhi H., Zhang K., He R., Su Y. (2024). Molecular understanding of the therapeutic potential of melanin inhibiting natural products. RSC Med. Chem..

[36] Tosstorff A., Kuhn B. (2025). Comparison of Molecular Recognition in Docking Versus Experimental CSD and PDB Data. J. Chem. Inf. Model..

[37] Zhang X., Shen C., Zhang H., Kang Y., Hsieh C.Y., Hou T. (2024). Advancing Ligand Docking through Deep Learning: Challenges and Prospects in Virtual Screening. Acc. Chem. Res..

[38] Lin X., Meng X., Lin J. (2023). The possible role of Wnt/beta-catenin signalling in vitiligo treatment. J. Eur. Acad. Dermatol. Venereol..

[39] Yu C.L., Pang H., Run Z., Wang G.H. (2025). Anti-Melanogenic Effects of L-Theanine on B16F10 Cells and Zebrafish. Molecules.

[40] Chin C.H., Chen S.H., Wu H.H., Ho C.W., Ko M.T., Lin C.Y. (2014). cytoHubba: Identifying hub objects and sub-networks from complex interactome. BMC Syst. Biol..

[41] Jia B.X., Huangfu Q.Q., Ren F.X., Jia L., Zhang Y.B., Liu H.M., Yang J., Wang Q. (2015). Identification and quantifica-tion of flavonoids and chromes in Baeckea frutescens by using HPLC coupled with diode-array detection and quadruple time-of-flight mass spectrometry. Nat. Prod. Res..

[42] Kamarazaman I.S., Ali N.A.M., Abdullah F., Saad N.C., Ali A.A., Ramli S., Rojsitthisak P., Halim H. (2022). In vitro wound healing evaluation, antioxidant and chemical profiling of Baeckea frutescens leaves ethanolic extract. Arab. J. Chem..

[43] Huong D.T.L., Xuan Duc D., The Son N. (2023). Baeckea frutescens L.: A Review on Phytochemistry, Biosynthesis, Synthesis, and Pharmacology. Nat. Prod. Commun..

[44] Nguyen T.H.T., Doan M.D., Tran D.T., Nguyen K.K., Nguyen T.H., Nguyen T.T., Nguyen T.T.T., Nguyen N.T. (2025). Optimization, chemical constituents and bioactivity of Baeckea frutescens L. essential oil extracted by micro-wave-assisted hydro-distillation. Plant Sci. Today.

