# Supplementary material for: Baeckea frutescens Suppresses Melanogenesis via Modulation of PKA/CREB and ERK/MAPK Pathways: Insights from Cellular, Zebrafish, and In Silico Analyses
Source: Molecules. 2026 May 16;31(10):1685. doi: 10.3390/molecules31101685 (PMC13209930; doi:10.3390/molecules31101685)
Supplement: Supplementary file 1 [file molecules-31-01685-s001.zip › molecules-4288625-supplementary.pdf]

Table S1: Number of deaths and mortality rates of zebrafish embryos (*n*=30).

Concentration (μg/mL)	Mortality Count (Fish)	Mortality Rate (%)	Phenotype
0	0	0	No obvious abnormalities
12.5	0	0	Similar to Normal Control
25.0	0	0	Similar to Normal Control
50.0	0	0	Similar to Normal Control
100	22	73	-
200	30	100	-

Table S2. UHPLC–Q Exactive Orbitrap-HRMS-based putative annotation of metabolites in the ethanolic extract of *B. frutescens* under positive and negative ion modes.

No.	Formula	Putative Annotation	Adduct	Retention Time (min)	Theoretical m/z	Observed m/z	Mass Error (ppm)	Fragmentation Score	Ion mode	Major MS/MS fragment ions (m/z)	Previous report/ reference
1	$C_5H_{12}O_5$	Arabinitol	M+H-H₂O	0.738	135.06577	135.06524	-3.924014	62.15	POS	55.9350, 57.0341, 69.0341, 72.9378, 90.9478, 99.0440, 135.0438, 135.9449, 136.0216, 153.9561	-
2	$C_5H_{11}NO_2$	DL-Norvaline	M+H	0.753	118.08625	118.08658	2.794568	59.77	POS	58.0659, 59.0738, 118.0867	-
3	$C_6H_{12}O_6$	Glucose	M+Na	0.754	203.05261	203.05248	-0.640228	60.8	POS	203.0527	-
4	$C_7H_{14}O_7$	Mannoheptulose	M+Na	0.774	233.06317	233.06327	0.429068	99.97	POS	233.0610	-
5	$C_6H_{12}O_5$	α-L-Rhamnose	M+Na	0.863	187.05769	187.05755	-0.748432	68.29	POS	55.9351, 116.9516, 117.9591, 118.9671, 119.9753, 134.9614, 163.9399, 163.9640, 181.9510, 187.0559	-
6	$C_7H_{14}O_7$	D-altrofurano-heptulose-3	M+H-H₂O	0.863	193.07125	193.07066	-3.055867	54.89	POS	69.0340, 83.0499, 93.0340, 95.0494, 111.0446, 121.0290, 129.0542, 139.0385, 147.0657, 157.0483	-
7	$C_{12}H_{22}O_{11}$	Turanose	M+Na	0.878	365.10543	365.10525	-0.493008	93.87	POS	185.0420, 203.0528, 365.1007	-
8	$C_6H_{14}O_5$	Fucitol	M+Na	1.133	189.07334	189.07347	0.687564	78.79	POS	189.0720	-
9	$C_7H_6O_5$	2,4,6-Trihydroxybenzoic acid	M+H-H₂O	6.099	153.01882	153.01845	-2.418003	68.82	POS	79.0181, 111.0449, 125.0232, 125.0593, 135.9453, 143.0330, 153.0169, 153.0291	-
10	$C_{14}H_{16}O_{10}$	3-Galloylquinic acid	M+H	6.106	345.08162	345.08155	-0.202851	86	POS	153.0188	-
11	$C_{15}H_{14}O_7$	Gallocatechin	M+H	7.809	307.08123	307.08051	-2.344656	66.23	POS	139.0384, 151.0382, 163.0393, 169.0489, 181.0504, 216.7670	-
12	$C_{14}H_{20}N_2O_3$	Feruloylputrescine	M+H	11.084	265.15467	265.15427	-1.508553	70.6	POS	58.9298, 91.3272, 104.5370, 135.7902, 139.9337, 145.0283, 177.0546, 216.7670, 248.1283, 265.1542	-
13	$C_{16}H_{18}O_9$	Cryptochlorogenic acid	M+Na	12.115	377.0843	377.08331	-2.625408	50.23	POS	216.7738, 359.0704, 377.0826	-
14	$C_{23}H_{28}O_{13}$	Picroside II	M+Na	18.044	535.14221	535.14396	3.270159	54.58	POS	143.2296, 153.0189, 215.0330, 233.0839, 263.0897, 292.7071, 305.1814, 415.1045, 535.1398	-

15	$C_{11}H_{12}O_4$	Sinapaldehyde	M+H	18.565	209.08083	209.0813	2.247934	57.55	POS	55.0187, 145.0282, 149.0584, 177.0549, 191.0699, 191.1437, 209.0617, 209.0812, 209.1169, 209.1526	-
16	$C_{15}H_{10}O_7$	6-Hydroxyluteolin	M+H	19.34	303.04993	303.04952	-1.352912	67.63	POS	303.0512, 304.0511	-
17	$C_{28}H_{36}O_{13}$	Episyringaresinol 4'-O-beta-D-glncopyranoside	M+NH₄	19.596	598.24941	598.2503	1.487674	68.76	POS	167.0695, 173.0606, 205.0842, 215.8173, 235.0954, 265.1077, 401.1528, 473.2330, 572.8181, 598.2349	-
18	$C_{15}H_{10}O_8$	Myricetin	M+H	19.869	319.04484	319.04458	-0.814932	68.14	POS	208.5368, 319.0449, 320.0448	[41-43]
19	$C_{21}H_{36}O_{10}$	A-D-Glucopyranoside	M+K	20.313	487.19401	487.19353	-0.985234	60.99	POS	487.1988, 488.1939	-
20	$C_{15}H_{10}O_6$	Scutellarein	M+H	20.371	287.05501	287.05444	-1.985682	83.46	POS	287.0521, 288.0573	-
21	$C_{21}H_{20}O_{10}$	Afzelin	M+H	20.371	433.11292	433.11373	1.870182	93.39	POS	71.0495, 85.0288, 129.0543, 287.0522, 288.0571	-
22	$C_{12}H_{14}O_5$	3,4,5-Trimethoxycinnamic acid	M+H-H_2O	20.66	221.08142	221.08126	-0.723715	52.24	POS	90.9767, 206.0565, 208.7245, 221.0800	-
23	$C_{16}H_{12}O_7$	Nepetin	M+H	20.695	317.06558	317.06593	1.103873	64.13	POS	317.0653, 318.0679	-
24	$C_{17}H_{14}O_6$	5,7-Dimethoxyluteolin	M+H	21.425	315.08631	315.08553	-2.475512	80.18	POS	315.0859, 316.0914	-
25	$C_9H_{14}O$	Isophorone	M+H	21.545	139.11174	139.11182	0.575077	93.52	POS	69.0340, 83.0500, 93.0706, 97.0653, 121.1007, 139.1108	-
26	$C_{16}H_{12}O_7$	3-O-Methylquercetin	M+H	21.645	317.06558	317.0649	-2.144667	56.28	POS	317.0653, 318.0674	-
27	$C_{15}H_{10}O_6$	Kaempferol	M+H	22.125	287.05501	287.05452	-1.70699	54.77	POS	208.5239, 287.0523, 288.0577, 305.1585	-
28	$C_{16}H_{12}O_6$	Diosmetin	M+H	22.39	301.07066	301.07075	0.298933	80.75	POS	301.0724, 301.1398, 302.0730	-
29	$C_{16}H_{12}O_7$	Tamarixetin	M+H	22.608	317.06558	317.06486	-2.270824	66.26	POS	317.0652, 318.0677	-
30	$C_{11}H_{16}O_2$	Dihydroactinidiolide	M+H	23.49	181.12231	181.12256	1.380283	90.31	POS	107.0863, 135.1165, 163.1109, 181.1210	-
31	$C_{11}H_{10}O_4$	Eugenin	M+H	23.718	207.06518	207.06531	0.627822	88.18	POS	207.0634	[43]
32	$C_{10}H_{18}O$	4-Carvomenthenol	M+H-H_2O	26.287	137.13307	137.13255	-3.791937	51.36	POS	59.7877, 81.0704, 89.7489, 91.6881, 95.0862, 137.0254, 138.0543	-
33	$C_{30}H_{46}O_5$	Alisol C	M+Na	27.241	509.32374	509.32322	-1.020962	65.33	POS	509.3240, 510.3261	-
34	$C_{10}H_{18}O$	1,8-Cineole	M+H-H_2O	27.301	137.13307	137.13253	-3.937781	51.95	POS	50.6433, 64.9364, 81.0704, 82.3722, 95.0862, 107.4272, 137.0237, 137.0599, 137.1323, 138.0543	[43]
35	$C_{12}H_{12}O_4$	Eugenetin	M+H	27.5	221.08083	221.08062	-0.949879	99.84	POS	221.0799	-

36	$C_{18}H_{36}O_2$	Methyl heptadecanoate	M+NH4	28.374	302.30535	302.30481	-1.786273	78.09	POS	302.3049, 303.3063	-
37	$C_{27}H_{28}N_2O_4$	Aurantiamide acetic acid	M+H	29.143	445.21218	445.21183	-0.786142	90.32	POS	91.0550, 105.0336, 117.0698, 134.0969, 194.1182, 224.1079, 252.0997, 445.2127	-
38	$C_{15}H_{24}O_2$	Neocurdione	M+Na	29.158	259.16685	259.16645	-1.543407	51.85	POS	129.0179, 214.4550, 259.1341, 259.1655, 260.1720	-
39	$C_{18}H_{14}O_3$	Dihydrotanshinone I	M+Na	29.623	301.08351	301.08322	-0.963188	66.29	POS	209.0195, 221.1171, 255.1237, 301.0842, 301.1402, 301.1792, 302.0849, 305.1756	-
40	$C_{12}H_{16}O_4$	Pogostone	M+H	30.326	225.11213	225.11225	0.533068	51.56	POS	179.1071, 192.0782, 207.1020, 225.1127	-
41	$C_{16}H_{14}O_4$	Pinostrobin	M+H	30.403	271.09648	271.09615	-1.217279	95.55	POS	131.0496, 167.0346, 271.0947, 272.0977	-
42	$C_{30}H_{46}O_4$	16alpha-Hydroxydehydrotrametenolic acid	M+H-H_2O	31.334	453.33691	453.33628	-1.389695	50.12	POS	187.1492, 189.1641, 201.1644, 210.3859, 210.4088, 389.3241, 407.3319, 435.3211, 453.3319, 454.3389	-
43	$C_{19}H_{20}O_3$	Cryptotanshinone	M+H	31.363	297.14852	297.1488	0.94229	92.49	POS	251.1437, 279.1350, 297.1507, 298.1487	-
44	$C_{20}H_{30}O_3$	Isosteviol	M+Na	31.415	341.20871	341.20618	-7.414817	53.62	POS	323.1290, 341.1427, 341.2068, 342.2108	-
45	$C_{15}H_{24}O$	Spathulenol	M+H-H2O	31.431	203.18002	203.17968	-1.673393	90.71	POS	81.0705, 95.0862, 109.1015, 119.0853, 133.1018, 147.1175, 161.1314, 175.1476, 203.1456, 203.1802	[44]
46	$C_{15}H_{24}O$	Alismol	M+H-H_2O	31.691	203.18002	203.17989	-0.639827	79.3	POS	95.0863, 105.0707, 107.0862, 109.1015, 119.0858, 133.1018, 147.1177, 185.1318, 203.1439, 203.1802	-
47	$C_{17}H_{16}O_4$	Flavokawain B	M+H	31.779	285.11213	285.11227	0.491035	85.66	POS	131.0497, 181.0502, 285.1138, 286.1142	-
48	$C_{30}H_{46}O_4$	Gypsogenin	M+H-H_2O	32.357	453.33691	453.33733	0.926463	58.42	POS	133.1018, 187.1492, 189.1642, 201.1644, 216.7944, 389.3242, 407.3320, 435.3214, 453.3313, 454.3389	-
49	$C_{15}H_{24}O$	Caryophyllene oxide	M+H-H_2O	32.762	203.18002	203.1799	-0.590609	88.4	POS	81.0704, 95.0864, 105.0701, 107.0861, 109.1016, 133.1000, 147.1174, 161.1319, 203.1456, 203.1802	[43]
50	$C_{15}H_{26}O$	Guaiol	M+H-H_2O	32.903	205.19567	205.19537	-1.462019	83.43	POS	57.0706, 81.0705, 93.0706, 95.0862, 109.1013, 121.1008, 149.0236, 149.1327, 205.1950, 209.3607	-
51	$C_{20}H_{20}O_4$	Licarin B	M+Na	33.083	347.12538	347.12492	-1.32517	50.57	POS	184.8906, 209.3574, 209.3801, 305.1584, 305.1928, 346.7905, 347.1243, 348.1259	-
52	$C_{18}H_{34}O_2$	Petroselinic acid	M+NH_4	33.588	300.2897	300.28967	-0.099904	78.88	POS	57.0706, 62.0607, 89.0599, 283.1750, 283.2613,	-

										300.2887, 301.1399, 301.2915	
53	$C_{30}H_{46}O_3$	Sanguisorbigenin	M+H-H_2O	34.128	437.342	437.34094	-2.423732	84.84	POS	81.0704, 95.0861, 189.1641, 201.1639, 203.1806, 239.1496, 285.1856, 391.3374, 437.3390, 438.3400	-
54	$C_{25}H_{43}NO$	N-Benzyloctadecanamide	M+H	37.791	374.34174	374.34194	0.534271	52.78	POS	150.0918, 374.2744, 374.3444, 375.3401	-
55	$C_{18}H_{32}O_{16}$	Gentianose	M+FA-H	1.062	549.16723	549.16834	2.021242	70.96	NEG	89.0233, 101.0235, 179.0561, 221.0657, 503.1571, 549.1694	-
56	$C_7H_{10}O_5$	Shikimic acid	M+FA-H	1.086	219.05102	219.05127	1.141287	58.01	NEG	93.0337, 99.9249, 111.0448, 116.9276, 129.0185, 155.0328, 173.0455, 174.8636, 189.8501, 218.8524	-
57	$C_7H_6O_5$	Gallic acid	M-H	3.642	169.01425	169.01333	-5.443328	94.42	NEG	125.0226, 169.0138	-
58	$C_7H_6O_4$	Protocatechuic acid	M-H	6.98	153.01933	153.01854	-5.162746	85.43	NEG	109.0285, 153.0192	-
59	$C_{15}H_{14}O_6$	(+)-Catechin hydrate	M-H	11.173	289.07176	289.07242	2.28317	86.1	NEG	109.0285, 125.0241, 205.0500, 211.4608, 245.0815, 289.0732	-
60	$C_{19}H_{30}O_8$	Roseoside	M+FA-H	13.69	431.19227	431.19355	2.968513	85.4	NEG	71.0126, 101.0235, 153.0905, 161.0451, 209.4123, 223.1351, 305.2756, 385.1880, 431.1947, 432.1963	-
61	$C_9H_{10}O_5$	Ethyl gallate	M-H	14.455	197.04555	197.04522	-1.67474	63.83	NEG	61.9874, 125.0241, 129.9743, 169.0140, 197.0211, 197.0449	[43]
62	$C_{34}H_{46}O_{18}$	Syringaresinol-di-O-glucoside	M+FA-H	16.54	787.26661	787.26831	2.15937	75.2	NEG	181.0509, 212.9478, 305.2813, 402.1317, 417.1552, 418.1636, 483.0792, 579.2142, 635.0887, 787.1013	-
63	$C_{15}H_{12}O_7$	Taxifolin	M-H	16.751	303.05103	303.05078	-0.824944	71.54	NEG	125.0241, 175.0400, 177.0185, 213.8784, 218.3399, 235.9250, 241.0537, 285.0417, 303.0473, 305.2642	-
64	$C_{22}H_{18}O_{10}$	Epicatechin gallate	M-H	16.983	441.08272	441.08288	0.362744	69.56	NEG	125.0241, 169.0143, 212.3519, 212.3718, 289.0739, 294.1796, 305.2814, 441.0822, 441.1709, 442.0876	-
65	$C_{14}H_6O_8$	Ellagic acid	M-H	17.06	300.99899	300.99937	1.262463	73.66	NEG	300.9973	-
66	$C_{21}H_{20}O_{12}$	Myricitrin	M-H	17.075	463.0882	463.08947	2.742458	83.82	NEG	271.0233, 316.0256, 317.0297, 463.0877, 464.0960	[43]
67	$C_{21}H_{20}O_{12}$	Isoquercitrin	M-H	17.629	463.0882	463.08931	2.396952	55.07	NEG	209.5653, 271.0253, 305.2756, 316.0253, 317.0286, 463.0877, 464.0947	-

68	$C_{21}H_{20}O_{13}$	Cannabiscitrin	M-H	17.908	479.08311	479.08448	2.859629	57.8	NEG	61.8007, 178.9973, 209.5588, 209.5849, 305.2527, 305.2871, 316.0259, 317.0274, 322.7610, 479.0809	-
69	$C_{20}H_{18}O_{11}$	Reynoutrin	M-H	18.208	433.07764	433.0782	1.293071	82.68	NEG	271.0252, 300.0293, 301.0364, 433.0756, 434.0833	-
70	$C_{20}H_{18}O_{11}$	Guaijaverin	M-H	18.502	433.07764	433.07782	0.41563	95.26	NEG	271.0254, 300.0295, 301.0368, 433.0759, 434.0844	-
71	$C_{20}H_{18}O_{11}$	Avicularin	M-H	18.907	433.07764	433.07789	0.577264	98.45	NEG	271.0252, 300.0293, 301.0364, 433.0756, 434.0834	-
72	$C_{21}H_{20}O_{11}$	Quercitrin	M-H	19.366	447.09329	447.09416	1.945903	98.22	NEG	271.0251, 300.0293, 301.0363, 447.0899, 448.0959	[43]
73	$C_{15}H_{12}O_{6}$	Dihydrokaempferol	M-H	19.389	287.05611	287.05669	2.020511	57.01	NEG	54.4334, 66.0134, 66.2079, 80.1748, 125.0241, 181.0565, 187.8467, 216.8442, 259.0620, 287.0560	-
74	$C_{21}H_{20}O_{12}$	Spiraeoside	M-H	19.572	463.0882	463.08926	2.288981	79.83	NEG	151.0035, 178.9973, 301.0363, 315.0145, 316.0252, 330.0425, 331.0472, 463.0878, 464.0953	-
75	$C_{10}H_{8}O_{4}$	Noreugenin	M-H	19.994	191.03498	191.03484	-0.73285	65.01	NEG	61.9870, 102.9477, 146.9370, 191.0353	-
76	$C_{20}H_{18}O_{10}$	Kaempferol 3-arabinofuranoside	M-H	20.181	417.08272	417.08379	2.565438	91.05	NEG	227.0366, 255.0309, 284.0318, 285.0418, 417.0821, 418.0896	-
77	$C_{22}H_{22}O_{11}$	Isorhamnetin 7-O-alpha-L-rhamnoside	M-H	20.688	461.10894	461.10939	0.975908	68.93	NEG	285.0421, 314.0450, 315.0515, 461.1147, 462.1154	-
78	$C_{15}H_{10}O_{6}$	Luteolin	M-H	21.052	285.04046	285.04056	0.350827	66.41	NEG	285.0419	-
79	$C_{15}H_{10}O_{7}$	Morin	M-H	21.052	301.03538	301.03594	1.860246	95.25	NEG	151.0032, 178.9975, 301.0368	-
80	$C_{15}H_{12}O_{5}$	Naringenin	M-H	21.799	271.0612	271.06182	2.287306	77.01	NEG	119.0500, 151.0033, 212.6579, 270.7382, 271.0636	-
81	$C_{36}H_{58}O_{10}$	Pedunculoside	M+FA-H	22.241	695.4012	695.4034	3.163641	80.01	NEG	61.9868, 487.3462, 488.3438, 649.3995, 695.4015, 696.4049	-
82	$C_{16}H_{14}O_{5}$	Poriol	M-H	24.056	285.07685	285.07744	2.069617	67.39	NEG	119.0495, 121.0292, 165.0191, 211.4807, 285.0788, 286.0834, 305.2585	-
83	$C_{30}H_{48}O_{6}$	Madecassic acid	M+FA-H	25.574	549.34329	549.34456	2.311851	60.91	NEG	208.3330, 305.2641, 503.3390, 504.3455	-
84	$C_{30}H_{48}O_{6}$	Terminolic acid	M+FA-H	25.792	549.34329	549.34455	2.293648	79.9	NEG	503.3376, 504.3460	-
85	$C_{15}H_{12}O_{4}$	Pinocembrin chalcone	M-H	25.796	255.06628	255.06658	1.176165	81.87	NEG	151.0029, 211.0756, 255.0661	-
86	$C_{15}H_{12}O_{4}$	Pinocembrin	M-H	26.374	255.06628	255.06655	1.058548	86.31	NEG	255.0660	-

87	$C_{30}H_{48}O_5$	Asiatic acid	M-H	28.4	487.3429	487.3444	3.077915	99.79	NEG	487.3480, 488.3433	-
88	$C_{16}H_{14}O_4$	Pinostrobin chalcone	M-H	29.445	269.08193	269.08191	-0.074327	90.7	NEG	165.0186, 226.0629, 254.0586, 269.0812	-
89	$C_{30}H_{48}O_5$	Bayogenin	M-H	29.56	487.3429	487.34445	3.180512	65.54	NEG	487.3481, 488.3447	-
90	$C_{18}H_{36}O_4$	9,10-Dihydroxystearic acid	M-H	30.513	315.25408	315.25486	2.474195	58.61	NEG	315.2555	-
91	$C_{30}H_{48}O_4$	23-Hydroxybetulinic acid	M-H	30.576	471.34798	471.34888	1.909417	76.78	NEG	471.3498, 472.3508	-
92	$C_{30}H_{48}O_4$	Hederagenin	M-H	31.091	471.34798	471.34868	1.485102	84.26	NEG	471.3496, 472.3522	-
93	$C_{30}H_{48}O_4$	Maslinic acid	M-H	31.681	471.34798	471.34877	1.676044	85.72	NEG	471.3497, 472.3518	-
94	$C_{30}H_{48}O_4$	Corosolic acid	M-H	31.97	471.34798	471.34873	1.591181	99.87	NEG	471.3497, 472.3513	-
95	$C_{30}H_{48}O_4$	Ganoderic acid DM	M-H	32.701	467.31668	467.31769	2.161275	52.39	NEG	176.4106, 216.8408, 421.3121, 467.3120, 468.3208	-
96	$C_{16}H_{32}O_3$	2-Hydroxypalmitic acid	M-H	33.878	271.22787	271.22853	2.433378	65.41	NEG	210.3363, 225.2225, 271.2268	-
97	$C_{30}H_{48}O_3$	Betulinic acid	M-H	34.22	455.35307	455.35516	4.589845	86.57	NEG	455.3533, 456.3565	[43]
98	$C_{30}H_{48}O_3$	Ursolic acid	M-H	34.466	455.35307	455.35509	4.436118	86.06	NEG	455.3533, 456.3569	[43]
99	$C_{30}H_{48}O_3$	Betulonic acid	M+FA-H	35.051	499.34289	499.34425	2.723579	67.02	NEG	212.7978, 453.3376, 454.3433	-
100	$C_{32}H_{50}O_4$	Ursolic acid acetic acid	M-H	36.12	497.36363	497.36445	1.648693	64.67	NEG	208.3685, 497.3653, 498.3667	-

Compound annotations were assigned based on accurate mass, retention time, MS/MS fragmentation matching, fragmentation scores, and spectral similarity using the LuMet-TCM database, consistent with MSI Level 2 putative annotation criteria. Major MS/MS fragment ions are listed when available. “-” indicates that previous occurrence in B. frutescens was not verified in the available literature.

Table S3. The parameters of the heated electrospray ionization-mass spectrometry (HESI-MS) of positive and negative ion model.

**Items**	**HESI +**	**HESI -**
Spray Voltage (V)	3800	-3200
Capillary Temperature (℃)	320	320
Aux gas heater temperature (℃)	350	350
Sheath Gas Flow Rate (Arb)	35	35
Aux gas flow rate (Arb)	8	8
S-lens RF level	50	50
Mass range (m/z)	100-1500	100-1500
Full ms resolution	60000	60000
MS/MS resolution	15000	15000
NCE/stepped NCE	10，20，40	10，20，40

Table S4. Instrument operation program of LC–MS analysis.

**Item**	**Parameter**
Column temperature	45°C
Flow rate	0.35 mL/min
Sample injection volume	2 μL
Mobile phase A	Water plus 0.1% formic acid
Mobile phase B	Acetonitrile

Table S5. Changes of solvents in gradient elution of LC–MS analysis.

**Time (min)**	**A%**	**B%**
0.0	100.0	0.0
3.0	100.0	0.0
18.5	80.0	20.0
20.0	65.0	35.0
26.0	60.0	40.0
35.0	5.0	95.0
38.0	5.0	95.0
38.1	100.0	0.0
40.0	100.0	0.0

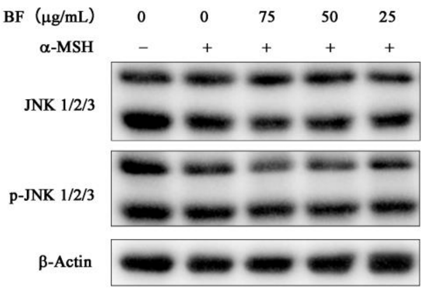

(a)

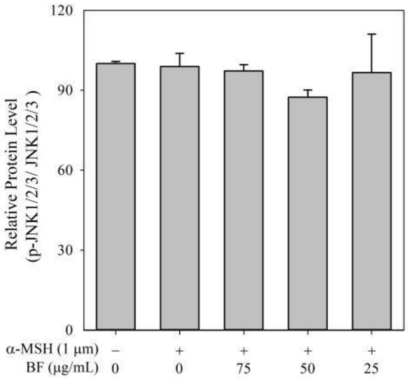

(b)

Figure S1. Effect of BF on JNK 1/2/3 MAPK signaling in B16F10 cells. (a) Western blot images showing the protein expression of JNK 1/2/3 and p-JNK 1/2/3. (b) Quantitative analysis of the p- JNK 1/2/3/ JNK 1/2/3. Data are presented as mean ± SD ($n = 3$). * $p < 0.05$ compared with α-MSH group.

Reference:

41. Jia, B.X.; Huangfu, Q.Q.; Ren, F.X.; Jia, L.; Zhang, Y.B.; Liu, H.M.; Yang, J.; Wang, Q. Identification and quantification of flavonoids and chromes in Baeckea frutescens by using HPLC coupled with diode-array detection and quadruple time-of-flight mass spectrometry. *Nat Prod Res* **2015**, *29*, 800-806, doi:10.1080/14786419.2014.987144.

42. Kamarazaman, I.S.; Ali, N.A.M.; Abdullah, F.; Saad, N.C.; Ali, A.A.; Ramli, S.; Rojsitthisak, P.; Halim, H. In vitro wound healing evaluation, antioxidant and chemical profiling of Baeckea frutescens leaves ethanolic extract. *Arabian Journal of Chemistry* **2022**, *15*, 103871.

43. Huong, D.T.L.; Xuan Duc, D.; The Son, N. Baeckea frutescens L.: A Review on Phytochemistry, Biosynthesis, Synthesis, and Pharmacology. *Natural Product Communications* **2023**, *18*, doi:10.1177/1934578x231189143.

44. Nguyen, T.H.T.; Doan, M.D.; Tran, D.T.; Nguyen, K.K.; Nguyen, T.H.; Nguyen, T.T.; Nguyen, T.T.T.; Nguyen, N.T. Optimization, chemical constituents and bioactivity of Baeckea frutescens L. essential oil extracted by microwave-assisted hydro-distillation. *Plant Science Today* **2025**, doi:10.14719/pst.8738.
